# Retraction and replacement of: A deep learning architecture for metabolic pathway prediction

**DOI:** 10.1093/bioinformatics/btae358

**Published:** 2024-07-29

This is a retraction and replacement of: Mayank Baranwal, Abram Magner, Paolo Elvati, Jacob Saldinger, Angela Violi, Alfred O Hero, A deep learning architecture for metabolic pathway prediction, *Bioinformatics*, Volume 36, Issue 8, April 2020, Pages 2547–2553, https://doi.org/10.1093/bioinformatics/btz954

It was brought to the author’s attention in October 2023 that the dataset used in this study included duplicates in the multi-class classification setting. The authors carefully reviewed and re-ran their experiments on the datasets with duplicates removed. In order to ensure the scientific accuracy of this paper, the text has now been revised, and we have replaced the original paper. The results of the paper remain unchanged. The new revised version has been reviewed and approved by the editors and is available at https://doi.org/10.1093/bioinformatics/btae359.

The authors have uploaded the de-duplicated datasets to the following Github page: https://github.com/baranwa2/MetabolicPathwayPrediction.

